# Diagnostic challenges and treatment approaches for *Clostridioides difficile* infection in IBD patients

**DOI:** 10.3389/fmicb.2026.1740387

**Published:** 2026-01-30

**Authors:** Asghar Ali, Khalid I. AlHussaini

**Affiliations:** 1 Clinical Biochemistry Lab, Department of Biochemistry, School of Chemical and Life Sciences, Jamia Hamdard, New Delhi, India; 2 Department of Internal Medicine, College of Medicine, Imam Mohammad Ibn Saud Islamic University (IMSIU), Riyadh, Saudi Arabia

**Keywords:** AMR, antimicrobial, CDI, FMT, immune dysregulation, microbiota, probiotics, therapy

## Abstract

**Background:**

*Clostridioides difficile* infection (CDI) poses a major clinical challenge in patients with inflammatory bowel disease (IBD) due to overlapping symptoms, diagnostic complexities, and distinct therapeutic considerations. The interaction between CDI and IBD involves disrupted gut microbiota, immune dysregulation, and disease-specific risk factors.

**Methods:**

This review critically examines the current evidence on the diagnosis and management of CDI in patients with IBD. Literature sources discussing diagnostic methodologies, therapeutic strategies, and preventive interventions were analyzed, with a focus on recent advances and their clinical applicability.

**Results:**

Diagnosing CDI in IBD remains difficult due to similar clinical presentations between infectious colitis and IBD flares, alongside limitations of stool assays, molecular tests, and endoscopic evaluations. Emerging diagnostic tools may enhance the accuracy and timeliness of detection. Standard therapies, antibiotics, and fecal microbiota transplantation (FMT) remain essential; however, their application requires individualization, taking into account immunosuppressive therapy, drug interactions, and the risk of recurrence. Treatment outcomes are further influenced by disease severity and patterns of antimicrobial resistance. Preventive strategies, including antimicrobial stewardship, probiotics, and vaccination, may help reduce the incidence of CDI among patients with IBD.

**Conclusion:**

CDI in IBD necessitates a personalized management approach that incorporates accurate diagnostics, targeted therapy, and preventive measures. Despite therapeutic advances, significant knowledge gaps persist regarding host microbiome interactions and the optimization of individualized treatment. Future research should focus on improving diagnostic precision and developing personalized medicine strategies to enhance outcomes for IBD patients affected by CDI.

## 1 Introduction

*Clostridioides difficile* infection (CDI) is a major cause of healthcare-associated diarrhea worldwide, usually triggered by antibiotic-induced disruption of the gut microbiota. *C. difficile*, a gram-positive, spore-forming anaerobe, produces toxins in the colon that can cause illness ranging from mild diarrhea to severe colitis, toxic megacolon or death (Smits et al., 2016; Czepiel et al., 2019). The pathogenesis of CDI develops when disruption of gut microbiota allows *C. difficile* to colonize the colon and release toxins. Two primary toxins, toxin A (TcdA) enterotoxin, which induces fluid secretion and mucosal injury, and toxin B (TcdB), which is more potent, damage the colonic epithelium. Certain strains also produce a binary toxin that may increase disease severity (Voth and Ballard, 2005; Aktories et al., 2017). The incidence of CDI has been increasing globally, particularly in developed countries, due to widespread use of antibiotics, aging populations and improved detection. Previously, due to limited healthcare settings, CDI is now emerging in the community, with cases reported among healthy people, younger adults and pregnant women (Lessa et al., 2015; Banawas, 2018). The disease causes high morbidity and mortality, particularly in the elderly, immunocompromised, and patients with croh (IBD) (Ananthakrishnan, 2011). Recurrent CDI (rCDI) is a common complication, occurring in approximately 20-30% of cases following initial treatment. Recurrence rates increase with each subsequent episode, complicating the clinical management and increasing healthcare burden (Johnson, 2009; McDonald et al., 2018). Accurate diagnosis is crucial but challenging due to variable test accuracy and overlapping symptoms with other gastrointestinal (GI) disorders, particularly IBD (Czepiel et al., 2019). Managing CDI in IBD patients is complex, as the coexistence of both inflammatory disorders necessitates individualized diagnostic and therapeutic strategies (Ananthakrishnan, 2011).

Patients with IBD face a four- to five-fold higher risk of CDI compared to the general population (Khanna, 2020b). This elevated susceptibility stems from frequent hospitalizations, immunosuppressive therapies, antibiotic use, gut microbiota disruption and compromised mucosal and immune defenses (Ananthakrishnan, 2011; Issa et al., 2007; Rodemann et al., 2007). The co-occurrence of CDI in patients with IBD represents a critical clinical scenario that poses unique diagnostic and therapeutic challenges. IBD, which primarily includes CD and UC, is characterized by chronic inflammation of the gastrointestinal tract, while CDI is an infectious disease that can cause severe colitis. When these two conditions overlap, their combined impact on patient health is often greater than the sum of their individual effects. The presence of CDI in IBD patients is linked to worse outcomes, including higher morbidity and mortality, hospitalization, and the need for surgery. It also increases the risk of complications like toxic megacolon and perforation, while recurrence demands repeated antibiotics that can aggravate IBD, promote resistance and disrupt gut microbiota (Dubberke and Olsen, 2012; McDonald et al., 2018).

As symptoms overlap with IBD flares, it is challenging to distinguish between the two. It requires careful evaluation for effective patient management. This can delay appropriate treatment and contribute to suboptimal patient outcomes. Moreover, standard diagnostic tools for CDI have limitations in sensitivity and specificity; therefore, there is a need for a tailored diagnostic approach to ensure timely and accurate diagnosis. Economically, this coexistence adds substantial costs due to prolonged hospitalizations, intensive care, frequent readmissions, extra diagnostics, aggressive therapies and surgical interventions (Dubberke and Olsen, 2012). Many IBD patients use immunosuppressive or immunomodulatory therapies that, while controlling disease activity, weaken immune defenses against infections like CDI. This increases the risk of severe or prolonged disease and recurrence. Corticosteroids are linked to worse outcomes and higher mortality, while biologics such as anti-TNF agents may also alter CDI severity by affecting immune response (Allegretti et al., 2016). The key strategies include improving diagnostics to distinguish flares from CDI, personalized treatment to control IBD while reducing the risk of CDI, using preventive measures like contact isolation precautions for patients infected with CDI, antimicrobial management, probiotics, and early risk identification. Collaboration between gastroenterologists, infectious disease specialists, and other healthcare providers is essential.

This review article aims to comprehensively explore the unique challenges and considerations involved in diagnosing and managing CDI in patients with IBD. We have discussed IBD, its pathophysiology, mechanism of development, and factors responsible for its development, as well as its major forms, UC and CD. We have also covered diagnosis and diagnostic challenges, conventional treatment strategies, challenges in treatment regimens, and preventive strategies. By examining the intersection of these two complex conditions, the review seeks to provide an in-depth understanding of the current landscape, identify gaps in existing knowledge, and highlight future directions for research and clinical practice.

## 2 Inflammatory bowel disease

IBD is a group of chronic, relapsing, and remitting disorders characterized by immune-mediated inflammation of the gastrointestinal (GI) tract. The two primary types of IBD are Crohn’s disease (CD) and ulcerative colitis (UC), each with distinct pathophysiological, clinical, and histological features (Ungaro et al., 2017).

### 2.1 Pathophysiology and diagnosis

The exact cause of IBD is unknown, but its etiology is multifactorial, involving genetic susceptibility, environmental influences, alterations in the gut microbiome, and immune dysregulation (Zhang and Li, 2014). Genetic studies have identified numerous risk loci, while factors like smoking, diet, infections and certain medications (e.g., nonsteroidal anti-inflammatory drugs, antibiotics) can trigger or worsen disease (Liu and Stappenbeck, 2016). IBD pathogenesis is strongly influenced by gut microbiome changes, with dysbiosis and reduced diversity commonly observed (Kostic et al., 2014). Abnormal regulation of both innate and adaptive immunity sustains the ongoing inflammation (Neurath, 2019).

Diagnosing IBD requires multimodal approach, including clinical evaluation, laboratory testing, endoscopy, histology and imaging studies (Magro et al., 2017). Laboratory tests may reveal elevated inflammatory markers, such as C-reactive protein (CRP) and fecal calprotectin, while colonoscopy provides direct visualization of the mucosa and biopsy for histological confirmation (Mosli et al., 2015; Magro et al., 2017). Imaging techniques like, computed tomography enterography (CTE), magnetic resonance enterography (MRE) and magnetic resonance imaging (MRI) of the pelvis are useful in assessing disease activity and complications like strictures, fistulas, and abscesses in CD (Panes et al., 2013). IBD management focuses on inducing and maintaining remission, improving quality of life and minimize complications. Treatments include aminosalicylates, corticosteroids, immunomodulators and biologics targeting specific inflammatory pathways. The choice of treatment depends on the severity and extent of disease, prior response, and the patient’s comorbidities (Ungaro et al., 2017).

### 2.2 Forms of IBD

#### 2.2.1 Ulcerative colitis

UC, a chronic disease, affects the colon and rectum, characterized by continuous mucosal inflammation that begins in the rectum and extends proximally in a uniform manner (Ungaro et al., 2017). The classification of the disease is based on the extent of disease: proctitis (rectum only), left-sided colitis (rectum and sigmoid colon), and Extensive colitis or pancolitis (entire colon). Typical symptoms include bloody diarrhea, urgency, tenesmus and abdominal cramping; but in severe forms, it may lead to toxic megacolon, perforation or an increased risk of colorectal cancer (Ananthakrishnan, 2015).

#### 2.2.2 Crohn’s disease

CD is a chronic inflammatory disorder that can affect any part of the GI tract, most commonly the terminal ileum and the colon. It is characterized by patchy, transmural inflammation (called “skip lesions”) that can cause deep ulcerations, fistulas and strictures (Torres et al., 2017). The symptoms often include abdominal pain, watery diarrhea, weight loss and fatigue (Baumgart and Sandborn, 2012). It may develop complications such as bowel obstruction, abscess formation, malnutrition, or extraintestinal manifestations including arthritis, skin lesions and eye inflammation (Roda et al., 2020).

## 3 Pathophysiology of CDI in IBD patients

### 3.1 Mechanisms of CDI development in the context of IBD

**Figure 1:**
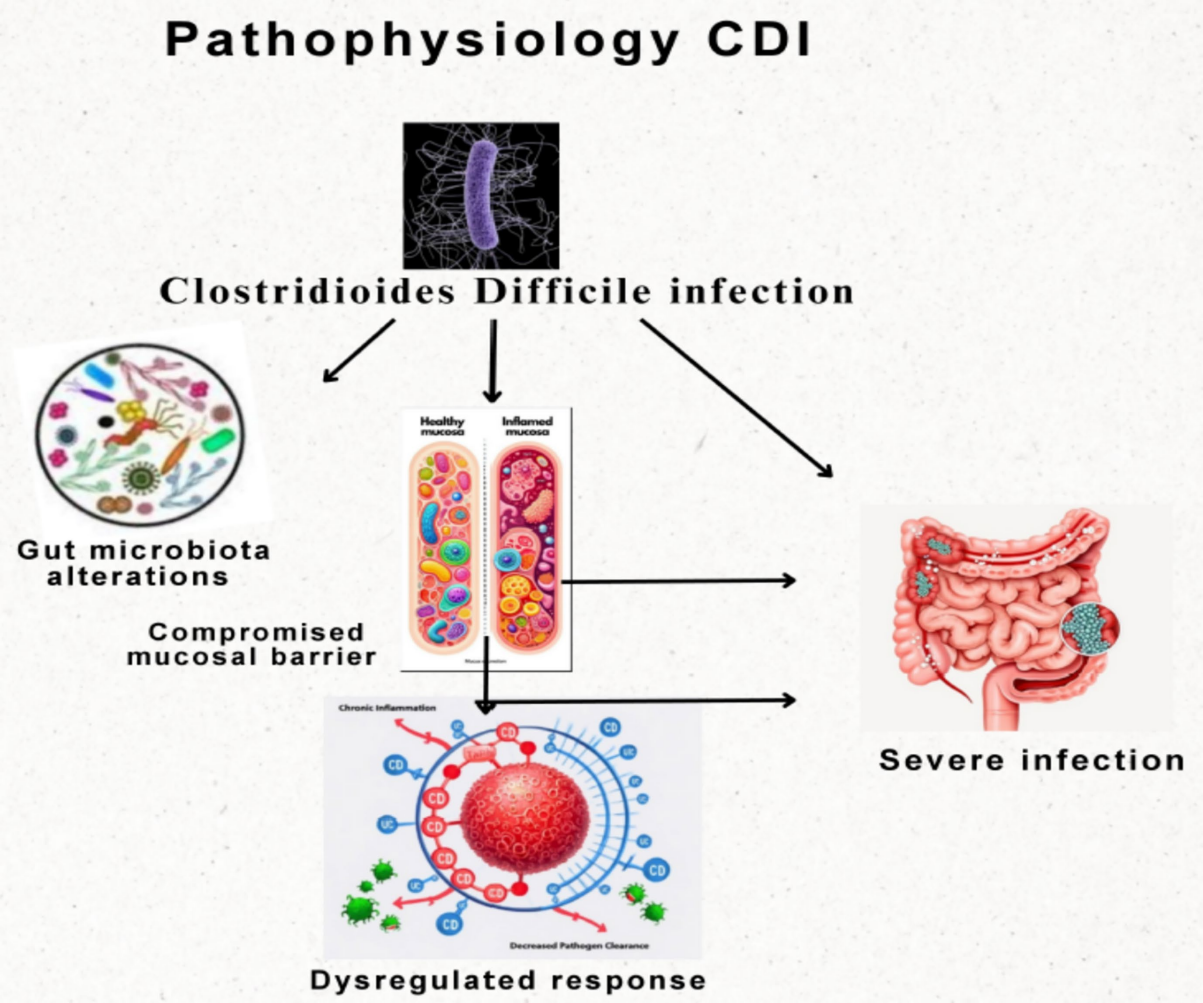
Mechanism of development of CDI.

CDI in IBD arises from a complex interplay of microbiological, immunological and therapeutic factors (Ananthakrishnan, 2011; Allegretti et al., 2016). Understanding these mechanisms is crucial for enhancing prevention and management in this high-risk group (Figure 1).

#### 3.1.1 Compromised mucosal barrier integrity

Active IBD is marked by chronic mucosal inflammation, transmural in CD and mucosal in UC, which increases intestinal permeability or “leaky gut.” This barrier dysfunction facilitates bacterial and toxin translocation, reduces protective mucus and antimicrobial peptide production and allows direct interaction of *C. difficile* toxins (TcdA, TcdB) with epithelial cells, amplifying inflammation and tissue injury (Hu et al., 2023).

#### 3.1.2 Dysregulated immune response

IBD involves chronic inflammation and dysregulation of the immune response against gut microbiota, driving persistent inflammation and increasing susceptibility to CDI (Ananthakrishnan, 2011; Razik et al., 2016). In CD, the immune response skews toward Th1/Th17, characterized by excess tumor necrosis factor-α (TNF-α), interferon-γ (IFN-γ), and interleukin-17 (IL-17), whereas UC exhibits a Th2 pattern with elevated interleukin-5 (IL-5) and interleukin-13 (IL-13). These imbalances promote inflammation, impair barrier repair and reduce clearance of *C. difficile* and its toxins. Immunosuppressive therapies, such as corticosteroids and biologics, further weaken host defenses, heightened CDI risk (Kurumi et al., 2024). Immunomodulators, such as azathioprine, mercaptopurine and methotrexate, inhibit t-cell proliferation and lower inflammatory responses, but also compromise immunity, particularly when combined with corticosteroids (Toruner et al., 2008; Lichtenstein et al., 2009). Biologics, such as anti-TNF agents (infliximab, adalimumab), integrin inhibitors (vedolizumab), and IL-12/23 inhibitors (ustekinumab), target specific pathways in moderate-to-severe IBD. While effective for disease control, they increase susceptibility to infections, including CDI, with anti-TNF agents, especially linked to opportunistic infections and impaired *C. difficile* clearance (Ananthakrishnan, 2011; Allegretti et al., 2016; Kirchgesner et al., 2018).

#### 3.1.3 Impact of IBD-related medications

Medications used in IBD, antibiotics, corticosteroids, immunosuppressants and biologics, can predispose to CDI. Antibiotics, especially clindamycin, cephalosporins, fluoroquinolones and broad-spectrum penicillins, disrupt gut microbiota and favor *C. difficile* overgrowth (Maharshak et al., 2018). Corticosteroids, used for flares, suppress local immunity, impair healing and linked to worse CDI outcomes. Immunosuppressants, such as azathioprine, methotrexate, and biologics (anti-TNF agents) further weaken host defenses, limiting pathogen clearance and increasing risk of severe or recurrent CDI (Boeriu et al., 2022).

#### 3.1.4 Genetic susceptibility

Genetic factors may also contribute to the increased risk of CDI in IBD patients. Specific genetic polymorphisms associated with IBD, such as those in the *NOD2* gene, which is involved in microbial recognition and immune response, may also predispose to an increased risk of infections, including CDI (Mousa et al., 2024). Variants in genes regulating the innate immune response, mucosal barrier function, and inflammatory pathways may further influence susceptibility to CDI and its severity in IBD patients (Zakerska-Banaszak et al., 2021).

#### 3.1.5 Host-microbiome interactions

The interaction between the gut microbiome and the immune system is central to CDI pathogenesis in IBD. Normally, the microbiome educates the immune system, ensuring tolerance to commensals and defense against pathogens. In IBD, this balance is lost, with dysbiosis and immune disruption that favor *C. difficile* growth and reduce protective metabolites like short-chain fatty acids (SCFAs), facilitating colonization and toxin production (Khanna, 2020a; Boeriu et al., 2022).

#### 3.1.6 Alterations in gut microbiota and mucosal immunity

A key factor contributing to the increased susceptibility to CDI in IBD patients is the disruption of gut microbiota, also known as dysbiosis. Generally, a diverse microbiome sustains intestinal homeostasis by competing with pathogenic microbes, by producing antimicrobial compounds and regulating the immune responses. In IBD, this balance is disturbed due to reduced Firmicutes and Bacteroidetes and increased level of Proteobacteria (Khan et al., 2019; Santana et al., 2022). The use of antibiotics and intestinal inflammation additionally worsen dysbiosis, creating environments that favor colonization of *C. difficile* by limiting competition and protective metabolites (Matsuoka and Kanai, 2015; Sultan et al., 2021). The interplay between gut microbiota and mucosal immunity is central to CDI pathogenesis, especially in IBD. In CD and UC, chronic inflammation disrupts gut ecology, causing dysbiosis and impaired immunity, which favor *C. difficile* colonization and increase CDI risk and severity (Spigaglia, 2016; Pandey et al., 2023). The gut microbiota is a diverse community of microorganisms essential for intestinal homeostasis, immune regulation, and protection against pathogens. In healthy individuals, it is dominated by Firmicutes and Bacteroidetes, which support nutrient metabolism, produce SCFAs and regulate host immune responses. These functions help maintain epithelial barrier integrity and limit pathogen overgrowth, including *C. difficile* (Dahal et al., 2023; Wiredu Ocansey et al., 2023). In IBD, this balance is disrupted, leading to dysbiosis. A key feature is reduced microbial diversity, which lowers gut resilience, decreases protective metabolite production and increases vulnerability to *C. difficile* colonization. Commensal bacteria such as *Faecalibacterium prausnitzii* and *Bacteroides fragilis* are notably depleted, leading to reduced SCFA levels, particularly butyrate. Butyrate is a vital energy source for colonocytes with strong anti-inflammatory and barrier-protective effects and its loss favors pathogen growth (Pandey et al., 2023; Wiredu Ocansey et al., 2023). At the same time, potentially harmful groups, such as Proteobacteria, including *E. coli,* often expand. These species exacerbate inflammation, produce toxic metabolites and compete with commensal organisms, further destabilizing the microbial ecosystem. Dysbiosis in IBD also disrupts metabolic functions, including bile acid transformation. The decrease in secondary bile acids removes another layer of colonization resistance, creating conditions that strongly favor *C. difficile* persistence and infection (Sinha et al., 2020; McMillan et al., 2024).

The GI mucosal immune system normally balances tolerance to commensals with pathogen defense; however, this equilibrium is disrupted in IBD, resulting in chronic inflammation, impaired barrier integrity, and increased susceptibility to CDI (Spigaglia, 2016; Kaur et al., 2023). Inflammation and mucosal erosion increase intestinal permeability, facilitating penetration of *C. difficile* spores and toxins and exacerbating disease severity (Zuo and Ng, 2018). Concurrent dysbiosis drives aberrant immune activation, excessive pro-inflammatory cytokine release, and loss of beneficial microbes, creating an oxidative, inflammatory gut environment that favors *C. difficile* colonization and persistence stress (Kostic et al., 2014; Lloyd-Price et al., 2019).

#### 3.1.7 Frequent antibiotic use

Antibiotics are a major risk factor for CDI, as they disrupt gut microbiota and reduce colonization resistance. IBD patients often require antibiotics for complications such as abscesses, perianal disease, pouchitis, or postoperative infections and frequently used agents like clindamycin, cephalosporins, fluoroquinolones, and broad-spectrum penicillins carry a particularly high risk (Issa et al., 2007; Khanna and Pardi, 2012). Broad-spectrum antibiotics profoundly reduce microbial diversity, eliminating protective bacteria and metabolites, weakening barrier integrity and allowing *C. difficile* spores to germinate and produce toxins. Repeated antibiotic exposure, common in IBD due to the relapsing disease course, causes cumulative microbiome damage, heightens susceptibility to CDI and may foster drug-resistant *C. difficile,* complicating treatment and recurrence (Carignan et al., 2008; Smits et al., 2016).

#### 3.1.8 Hospitalization and healthcare exposure

IBD patients often face repeated hospitalizations for flares, complications or surgery, which significantly raise the risk of CDI. Healthcare settings contain abundant *C. difficile* spores and prolonged stays, frequent admissions or ICU exposure increase vulnerability to hospital-acquired infection through contaminated surfaces, equipment or staff (Khanna and Pardi, 2012; Leffler and Lamont, 2015). Additionally, many IBD patients receive proton pump inhibitors (PPIs) for reflux or related symptoms. By reducing gastric acidity, PPIs promote survival and colonization of ingested spores, further heightening CDI risk, especially in already hospitalized patients (Janarthanan et al., 2012; Deshpande et al., 2013).

#### 3.1.9 Severity of underlying IBD

The severity and extent of IBD are important risk factors for CDI, with patients who have extensive colitis or pancolitis at particularly high risk (Ananthakrishnan, 2011; Razik et al., 2016). During active flares, inflammation compromises the mucosal barrier and impairs local immune responses, allowing *C. difficile* spores and toxins to penetrate more easily. Surgical interventions, such as colectomy or bowel resection, further disrupt gut architecture and defenses, predisposing to infection. Patients with an ileal pouch, particularly after colectomy for UC, are especially prone to *C. difficile* associated pouchitis, a difficult complication to manage (Ghoshal et al., 2024).

#### 3.1.10 Nutritional deficiencies

Nutritional deficiencies are frequent in IBD due to malabsorption, inflammation and diet restrictions. Deficits in key nutrients, such as vitamin D and zinc, weaken immune defenses and mucosal integrity, thereby increasing the risk of infection. In particular, vitamin D deficiency, commonly found in IBD, impairs antimicrobial immune function and barrier protection, predisposing patients to CDI (Ananthakrishnan et al., 2012; Jørgensen et al., 2013).

## 4 Diagnostic challenges in identifying CDI in IBD patients

### 4.1 Overlapping clinical features of CDI and IBD

Distinguishing CDI from an IBD flare is challenging because both share symptoms such as diarrhea, abdominal pain, fever and increased stool frequency. This overlap complicates diagnosis and when CDI coexists with IBD, it is associated with worse outcomes, including higher morbidity, more hospitalizations and increased surgical risk. Diarrhea is common to both; in CDI it is often watery, profuse and foul-smelling, while in IBD it varies in consistency and may contain blood or mucus (Ananthakrishnan, 2011). Abdominal pain in CDI is often diffuse, whereas in IBD it reflects inflammation sites (e.g., RLQ in Crohn’s, LLQ in UC), though both can overlap. Fever occurs with CDI as a sign of infection but may also signal severe IBD or complications like abscesses. Leukocytosis is typical of CDI (often >15,000/mm^3^) but also occurs in severe IBD, limiting diagnostic value. Urgency and tenesmus are hallmarks of UC but may also appear in CDI due to toxin-induced colonic inflammation. Finally, fatigue and malaise, secondary to inflammation, electrolyte loss or anemia, are nonspecific and common to both conditions (Alhobayb and Ciorba, 2023). This overlap leads to underdiagnosis, allowing CDI to progress to severe complications such as toxic megacolon, perforation or sepsis. The challenge is compounded in IBD patients on immunosuppressive therapy, as agents like corticosteroids and biologics can dull the inflammatory response, producing atypical or milder CDI symptoms that mimic a flare and further delay recognition (Goodhand et al., 2011; Khanna and Pardi, 2012).

Misdiagnosing CDI as an IBD flare can lead to inappropriate use of corticosteroids or immunosuppressants, worsening infection, while mistaking a flare for CDI and using antibiotics may aggravate dysbiosis and IBD activity (Ananthakrishnan, 2011; Goodhand et al., 2011). Such errors, along with diagnostic delays, increase morbidity and mortality, with CDI in IBD patients linked to longer hospital stays, more surgeries, and higher death rates. Accurate distinction requires a comprehensive workup, including stool toxin or PCR testing and, in some cases, endoscopy or imaging, especially when symptoms overlap or responses to standard therapy are poor (Surawicz et al., 2013; Khanna and Pardi, 2014).

### 4.2 Limitations of current diagnostic methods

Timely diagnosis of CDI in IBD is essential but difficult because its symptoms closely resemble IBD flares. Current diagnostic tools, including stool assays, cultures, PCR, toxin tests, and radiologic or endoscopic evaluations, each have limitations in accuracy, speed, and applicability in IBD, making differentiation challenging.

#### 4.2.1 Stool testing and microbiological culture

Stool toxin testing is widely used to diagnose CDI by detecting TcdA and TcdB, the main virulence factors. However, enzyme immunoassays (EIAs) have limited sensitivity, especially in IBD patients with low toxin levels or dilute stools, leading to false negatives and delayed treatment (Planche et al., 2008; Khanna and Pardi, 2014). Microbiological culture of *C. difficile,* followed by toxin confirmation, is considered the diagnostic “gold standard” due to high sensitivity. Yet, it is slow (48-96 h), labor-intensive and requires specialized expertise. Cultures may also yield false positives by detecting non-toxicogenic strains, risking unnecessary treatment, which limits routine clinical utility (Kuijper et al., 2006; Surawicz et al., 2013).

#### 4.2.2 Polymerase chain reaction

Polymerase chain reaction (PCR) Testing detects *C. difficile* toxin genes *TcdA* and *TcdB* with high sensitivity and rapid results but carries notable limitations in IBD (Khanna and Pardi, 2014; Crobach et al., 2016). It identifies gene presence regardless of toxin activity often detecting asymptomatic colonization common in IBD patients with frequent healthcare exposure. Thus, PCR may lead to overdiagnosis and overtreatment. In addition, it cannot distinguish CDI from an IBD flare since both cause similar GI symptoms (Goodhand et al., 2011; Khanna and Pardi, 2014). PCR is also costly, resource-intensive and requires specialized equipment. Multistep algorithms, combining a sensitive test (PCR or GDH) with a specific toxin assay (EIA), improve accuracy but add complexity. This approach can delay treatment in urgent cases and sometimes produces conflicting results, such as PCR-positive/toxin-negative finding. In IBD patients, such ambiguity increases the risk of both under- and over-diagnosis, complicating management decisions (Surawicz et al., 2013; Khanna and Pardi, 2014).

#### 4.2.3 Radiologic and endoscopic assessments

Radiologic Imaging techniques, such as computed tomography (CT) or magnetic resonance imaging (MRI), can assess the severity and complications of CDI, including toxic megacolon or perforation; however, their findings (wall thickening, bowel dilation, and mucosal changes) overlap with those of IBD flares, thereby limiting their diagnostic value. CT also involves radiation, a concern in IBD patients requiring repeated imaging, especially younger individuals (Guerri et al., 2019). Imaging is thus reserved for severe cases where complications are suspected, rather than routine evaluation of mild or moderate disease. Endoscopic evaluation (colonoscopy, sigmoidoscopy) provides direct mucosal visualization and may help distinguish CDI from IBD (Wilcox, 2012). However, it carries significant risks in severe colitis, including perforation due to fragile, inflamed mucosa. Endoscopic findings are often non-specific, as pseudomembranes, erythema and ulceration may appear in both conditions, while typical IBD features, such as continuous inflammation or crypt abscesses, can sometimes mimic CDI (Surawicz et al., 2013; Lembo et al., 2016). Endoscopy also requires specialized resources, is costly, and may not be feasible in unstable or critically ill patients, limiting its use in urgent diagnostics.

#### 4.2.4 Role of biomarkers

Biomarkers and emerging diagnostic tools present opportunities to improve CDI diagnosis in IBD, where overlapping symptoms and limitations of conventional methods (stool tests, PCR, imaging) complicate differentiation from flares (Khanna and Pardi, 2014; Crobach et al., 2016). Advances in biomarker discovery and novel approaches may enhance accuracy, enable earlier detection and support more tailored management (Ananthakrishnan, 2011; Surawicz et al., 2013). Biomarkers are measurable indicators that can provide insight into disease activity, infection, or treatment response. In IBD patients, distinguishing a flare from CDI is challenging and biomarkers may help guide further evaluation (Ananthakrishnan, 2011; Khanna and Pardi, 2014). Fecal calprotectin is a commonly used marker of mucosal inflammation, released by neutrophils during intestinal injury. While sensitive for detecting inflammation, it is non-specific (levels rise in both IBD flares and CDI), so it cannot differentiate between the two. Its value lies in identifying patients who require additional testing, such as stool toxin assays or PCR (Crobach et al., 2016). Fecal lactoferrin, another neutrophil-derived protein, behaves similarly. Elevated in both IBD and CDI, it confirms intestinal inflammation but does not clarify the cause, limiting its utility for discrimination (Zhou et al., 2014). C-Reactive Protein (CRP) reflects systemic inflammation and is widely used in IBD monitoring. Like fecal markers, CRP rises in both flares and CDI, lacking specificity but serving as a useful adjunct when interpretated alongside stool tests and clinical data (Mosli et al., 2015). Serum procalcitonin is more specific to bacterial infections and may be elevated in CDI compared to IBD flares. However, sensitivity and specificity vary and evidence remains limited (Shindo et al., 2017). More studies are needed to establish its diagnostic role in IBD-associated CDI. Fecal biomarkers like calprotectin and Lactoferrin are commonly used to assess intestinal inflammation in IBD but lack specificity, as both IBD flares and CDI elevate these markers, limiting diagnostic value.

#### 4.2.5 Emerging diagnostic tools

Emerging diagnostic approaches, including molecular methods, immunoassays, and point-of-care tests, aim to overcome the limitations of conventional diagnostics for CDI, especially in complex cases involving IBD. These tools promise faster, more accurate and more specific diagnosis (Burnham and Carroll, 2013; Crobach et al., 2016). Multiplex PCR panels can simultaneously detect multiple gastrointestinal pathogens in one stool sample, providing rapid and highly sensitive results. This is valuable in IBD patients, where co-infections can mimic flares. However, like standard PCR, they detect toxin genes rather than active toxin production, raising the risk of false positives in asymptomatic carriers (Khanna and Pardi, 2014; Tamma et al., 2019). Quantitative real-time PCR (qPCR) offers an estimate of bacterial load by quantifying toxin gene copy numbers, potentially correlating with disease severity and helping distinguish active CDI from colonization. Yet, it still cannot directly assess toxin activity and requires further validation (Lessa et al., 2015; Crobach et al., 2016). Refined enzyme immunoassays (EIAs) for TcdA and TcdB have been developed to improve sensitivity and capture low levels of toxin, reducing false negatives seen with older EIAs. Despite progress, sensitivity and specificity remail imperfect (Planche et al., 2008). Toxin activity assays, which measure cytotoxic effects of toxins on cultured cells, provide direct evidence of pathogenic toxin production and may differentiate colonization from true infection. However, they are technically demanding slow, and not widely available for routine use (Crobach et al., 2016). Host immune response assays represent another novel approach, measuring cytokines, chemokines and other biomarkers elevated during CDI. Markers such as IL-8 or fecal lactoferrin may help distinguish CDI from IBD flares, but further studies are requiring before clinical adoption (Khanna et al., 2015; Singh et al., 2015).

Next,-generation sequencing (NGS) and microbiome analysis allow detailed assessment of gut microbial communities and metabolic signatures. These techniques can reveal CDI-related dysbiosis distinct from IBD activity, offering new diagnostic insights. At present, however, they remain costly, time-intensive and mainly investigational (Seekatz et al., 2014; Guh and Kutty, 2018). Point-of-care (POC) tests using lateral flow immunoassays to detect toxins or glutamate dehydrogenase (GDH) antigen provide rapid bedside results. This quick turnaround can guide timely therapy in high-risk IBD patients. Limitations remain in variable sensitivity and specificity and validation in larger IBD cohorts is still needed. In summary, while conventional stool assays, PCR and imaging have notable drawbacks, emerging molecular and immune-based tools offer promising advances. Although several remain experimental, their continued refinement could significantly improve the timely and accurate differentiation of CDI from IBD flares, leading to better outcomes in this vulnerable population (Burnham and Carroll, 2013; Seekatz et al., 2014; Crobach et al., 2016).

## 5 Treatment approaches for CDI in IBD patients

### 5.1 Standard therapies for CDI

Managing CDI in IBD patients is challenging due to overlapping symptoms and the heightened risk of morbidity and mortality when both conditions coexist. Standard CDI therapies include antibiotics and fecal microbiota transplantation (FMT), which aim to eradicate *C. difficile* and restore microbial balance. In IBD patients, however, these treatments require special consideration given their underlying disease activity and concurrent immunosuppressive therapies. This section reviews the mechanisms, effectiveness, and special considerations for applying standard CDI treatments in IBD patients (Crobach et al., 2016; Guh and Kutty, 2018).

### 5.2 Antibiotics for CDI

Antibiotic therapy remains the cornerstone of CDI management, with treatment choices guided by disease severity, prior therapies and comorbidities such as IBD. The main antibiotics are metronidazole, vancomycin, and fidaxomicin (Cornely et al., 2012; McDonald et al., 2018). Metronidazole was once a first-line therapy for mild CDI but is now discouraged as primary treatment due to lower efficacy versus vancomycin or fidaxomicin, especially in severe or recurrent cases. It inhibits DNA synthesis in anaerobes but achieves lower colonic concentrations. In IBD patients, altered microbiota and reduced absorption can further limit effectiveness, while side effects such as neuropathy or GI intolerance may add risk. Nowadays, metronidazole is largely reserved for mild cases when cost or access limits preferred treatments, or occasionally in combination therapy, though evidence is limited (Khanna et al., 2015; Tamma et al., 2019). Vancomycin is now the preferred first-line treatment mild to severe CDI, given orally to deliver high colonic concentrations with minimal systemic absorption. It inhibits bacterial cell wall synthesis and is highly effective, including in IBD patients, where it shows higher cure rates and fewer recurrences compared to metronidazole (Cornely et al., 2012; McDonald et al., 2018). Standard dosing is 125 mg orally four times daily for 10 days, with higher doses used in severe cases. Recurrent or severe IBD cases may require prolonged or repeated courses, but monitoring is essential to minimize resistance risk (Debast et al., 2014). Fidaxomicin is a newer macrocyclic antibiotic with cure rates at least comparable to vancomycin and a significantly lower recurrence rates (Louie et al., 2011; Cornely et al., 2012). It inhibits bacterial RNA synthesis and due to its narrow spectrum against *C. difficile*, preserves normal gut microbiota, which is an important advantage for IBD patients at high risk of relapse. Clinical data support its use in recurrent CDI, with reduced disruption of gut flora aiding IBD stability. The standard regimen is 200 mg twice daily for 10 days. Its major limitation remains cost, though guidelines increasingly recommend it for IBD patients with frequent relapses (Khanna et al., 2015; Guh and Kutty, 2018).

### 5.3 Fecal microbiota transplantation

FMT involves transferring stool from a healthy donor to restore gut microbial balance in patients with recurrent CDI. It is highly effective, with cure rates exceeding 80-90% especially when standard antibiotics fail (van Nood et al., 2013; Kelly et al., 2014). FMT restores colonization resistance by reintroducing a diverse microbiome that outcompetes *C. difficile* and supports gut health. Delivery routes include colonoscopy, nasogastric or nasojejunal tube, enema or oral capsules (Cammarota et al., 2015; Allegretti et al., 2020). In IBD patients, FMT demonstrates high efficacy for treating CDI but raises unique considerations assome patients experience IBD flares post-FMT, whereas others maintain or even improve disease stability (Cammarota et al., 2015; Allegretti et al., 2020). MT is reserved for IBD patients with multiple CDI recurrences unresponsive to antibiotics, though it is being considered earlier in difficult cases given their higher recurrence risk (Kao et al., 2017; Quraishi et al., 2017). Colonoscopy allows targeted delivery but carries higher procedural risks in severe colitis. Less invasive methods, such as capsules or enemas, are safer alternatives though efficacy may vary (Costello et al., 2019; Allegretti et al., 2020).

### 5.4 Concurrent IBD therapies

Many IBD patients receive immunosuppressants, such as corticosteroids, thiopurines, or biologics, which increase the risk of severe or recurrent CDI and complicate antibiotic selection. Close coordination between gastroenterology and infectious disease specialists is essential to balance efficacy and safety (Kelly et al., 2014; Khanna et al., 2015). Both antibiotics and FMT may trigger IBD flares as Antibiotics can disrupt the gut microbiota, while FMT, may provoke an immune response that worsens IBD. Hence, Careful monitoring and individualized therapy are necessary to minimize these risks (Allegretti et al., 2016; Saha et al., 2021). As IBD patients are highly susceptible to recurrent CDI, a proactive treatment strategy is needed. Fidaxomicin is often preferred for its lower recurrence rates, while FMT is considered in patients with multiple relapses to prevent further episodes and reduce disease burden (Cornely et al., 2012; Louie et al., 2011).

### 5.5 Considerations for immunosuppressive and biologic agents

Immunosuppressive and biologic therapies are central to IBD management but complicate CDI treatment by altering immune responses and gut microbiota. Management requires balancing infection eradication with inflammation control (Kelly et al., 2014; Khanna et al., 2015). Corticosteroids agents like prednisone and budesonide are widely used for flares but impair host defenses and worsen CDI outcomes. When CDI is diagnosed, tapering or reducing steroids is generally advised to lower the risk of severe or recurrent infection. If tapering threatens IBD stability, the lowest effective dose should be used, with consideration of alternatives such as biologics or cyclosporine (Allegretti et al., 2016; Saha et al., 2021). Immunomodulator drugs (azathioprine, methotrexate, thiopurines) maintain remission but increase susceptibility to infections. In mild CDI, they may be continued with close monitoring. In severe or fulminant disease, temporary discontinuation may help clear the infection, although the risk of flare must be weighed carefully. Individualized decisions and coordination between gastroenterology and infectious disease specialists are essential (Khanna and Pardi, 2012; Allegretti et al., 2016).

Biologic Agents (anti-TNF, integrin inhibitors, IL-12/23 inhibitors) are highly effective for moderate to severe IBD, biologics also raise CDI risks. In mild CDI with stable patients, biologics can often be continued while treating the infection. However, in severe CDI sepsis, therapy should be paused until stabilization. Switching to agents with gut-selective activity, such as vedolizumab may reduce systemic immunosuppression while maintaining IBD control (Kelly et al., 2014; Khanna et al., 2015; Saha et al., 2021).

### 5.6 Management of recurrent CDI

Recurrent CDI is common in IBD patients, affecting up to 30% after initial treatment and more in those with multiple prior recurrences. Management requires a proactive, individualized approach that accounts for their higher recurrence risk and underlying disease (Cammarota et al., 2015; Allegretti et al., 2016). Extended or pulse regimens of vancomycin or fidaxomicin are frequently used to reduce relapse. These schedules gradually decrease *C. difficile* burden while supporting microbiota recovery, which is vital for restoring colonization resistance (Kelly et al., 2014). Owing to its narrow spectrum and reduced effect on commensal flora, fidaxomicin is increasingly favored for recurrent CDI. For IBD patients, who are particularly prone to relapse or flares from broad-spectrum agents, fidaxomicin is often the optimal choice, especially in those with multiple recurrences (Louie et al., 2011; Cornely et al., 2012; Allegretti et al., 2016).

FMT is highly effective for recurrent CDI after antibiotic failure, achieving cure rates exceeding 80–90% in most studies. Restoring microbial diversity strengthens colonization resistance against *C. difficile.* In IBD patients, however, FMT requires consideration; while many respond well, some experience disease exacerbations (Cammarota et al., 2015; Allegretti et al., 2016). FMT is generally reserved for IBD patients with multiple CDI recurrences who do not respond to antibiotics. Delivery routes include colonoscopy, oral capsules, or enemas, with the choice based on severity and safety considerations. Colonoscopic delivery allows targeted placement but carries procedural risks, especially in severe colitis. Given potential flare risk, FMT should be performed in experienced centers, with individualized planning and post-procedure monitoring (Kelly et al., 2014; Allegretti et al., 2016; Saha et al., 2021).

### 5.7 Role of surgery in severe cases

Surgical intervention may be required in IBD patients with severe or fulminant CDI who fail medical therapy or develop life-threatening complications such as toxic megacolon, perforation, or refractory colitis (Hall and Mah, 2017; Barkun et al., 2019). Surgery is reserved for patients who do not improve with optimized medical management, including high-dose antibiotics and supportive care. Common triggers include clinical deterioration, toxic megacolon, perforation, severe bleeding, or drug-refractory colitis. Subtotal or total colectomy is the standard, often lifesaving in fulminant colitis with perforation, peritonitis, or sepsis. In selected cases, a diverting loop ileostomy with colonic lavage may be considered as a less invasive alternative, offering colonic preservation while reducing toxins, though suitability depends on disease extent and patient stability (Siddiqui and Young, 2020). Outcomes in IBD patients are often complicated by malnutrition, immunosuppression, and comorbidities. Surgical decisions should be made within a multidisciplinary team, including gastroenterologists, surgeons, and infectious disease specialists, to optimize preoperative care and improve prognosis (Steele et al., 2015).

### 5.8 Treatment guidelines

Diagnostic guidelines, both the IDSA and ACG guidelines endorse a stepwise diagnostic approach for *Clostridioides difficile* infection, in which nucleic acid amplification testing or glutamate dehydrogenase screening is used initially, followed by toxin enzyme immunoassay to confirm active toxin production. The ACG further recommends repeat testing in patients with negative initial results when clinical suspicion remains high.

The guidelines from the Infectious Diseases Society of America (IDSA) and the Society for Healthcare Epidemiology of America (SHEA) guidelines recommend oral vancomycin (125 mg four times daily for 10 days) as first-line therapy for mild to non-fulminant CDI, while fulminant disease is treated with high-dose oral vancomycin (500 mg four times daily) plus intravenous metronidazole, with adjunctive rectal vancomycin in cases of severe ileus. For a first recurrence, a pulsed–tapered vancomycin regimen is advised. Despite therapy, approximately 25% of patients relapse; however, prolonged vancomycin courses (21–42 days) significantly reduce recurrence compared with shorter regimens (10–14 days). For recurrent *CDI,* the 2017 IDSA guidelines recommend rifaximin (400 mg three times daily for 20 days) following a 10-day course of vancomycin, in addition to tapering or pulse regimens with vancomycin. The rifaximin “chaser” strategy has shown promise in reducing recurrence and is included in current IDSA guidance for second or subsequent CDI episodes.

The 2019 British Society of Gastroenterology guidelines for IBD state that discontinuation of corticosteroid therapy during treatment for CDI is not required in patients with acute severe UC. Probiotics are not recommended for the prevention of *CDI*, and the American College of Gastroenterology guidelines explicitly advise against their use for CDI prophylaxis. The 2021 European Crohn’s and Colitis Organization (ECCO) guidelines on IBD management indicate that the impact of immunosuppressive therapies on the course and progression of CDI in patients with IBD remains uncertain. The 2018 guidelines jointly issued by the British Society of Gastroenterology and the Healthcare Infection Society recommend the use of FMT for patients with recurrent CDI who have experienced at least two recurrences, as well as for those with a single recurrence who are considered at high risk for subsequent episodes, including individuals with severe or complicated CDI.

## 6 Challenges in treating CDI in the context of IBD

### 6.1 Impact of IBD severity and flare-ups on CDI treatment

The severity of IBD and the presence of flares strongly influence the management of CDI. Treatment is complicated by active intestinal inflammation, the immunosuppressive therapies often required for IBD, and the disrupted gut microbiota that increases susceptibility to CDI. Disease severity and flare activity can affect diagnosis, therapeutic response and overall prognosis in this high-risk population (Khanna and Pardi, 2012). Severe IBD flares share symptoms with CDI, such as diarrhea, abdominal pain, rectal bleeding, fever, and leukocytosis, making clinical distinction difficult. This overlap can delay diagnosis, lead to inappropriate therapy, or postpone CDI-specific treatment and worsen outcomes. Active colitis with compromised mucosa may further mask or mimic CDI, which may complicate the diagnostic process (Khanna and Pardi, 2012). Patients with severe IBD or active flares are more prone to fulminant CDI due to disrupted mucosa, loss of protective gut flora, and immune dysregulation that limits effective response to infection. These patients face higher risks of serious complications requiring prompt, aggressive and often multidisciplinary management (Martínez-Lozano et al., 2025). Managing CDI in IBD patients is complicated by inflammation-driven changes in absorption, motility and pharmacokinetics. During severe flares, oral antibiotics like vancomycin may achieve unpredictable or subtherapeutic levels due to mucosal damage, sometimes requiring higher or adjusted dosing. Patients are also more prone to antibiotic-related side effects, including GI intolerance and secondary infections, further complicating therapy (Debast et al., 2014). Recurrence is more frequent in IBD, affecting up to 40–60% of patients with severe or extensive disease, leading to greater morbidity and healthcare burden. Extended or pulsed regimens of fidaxomicin or vancomycin may also help suppress *C. difficile* while allowing gradual microbiome recovery (Cornely et al., 2012). FMT is highly effective in the general population (80-90% cure rates), but outcomes in IBD are less predictable. Thus, FMT should be reserved for carefully selected IBD patients, with pre-procedure optimization and close follow-up (Quraishi et al., 2017).

### 6.2 Immunosuppressive therapy and CDI management

IBD patients often require immunosupression but this can hinder *C. difficile* clearance and increase recurrence risk (Khanna and Pardi, 2012; Torres et al., 2020). In mild CDI, immunosuppressive therapy may be continued with close monitoring. For severe disease, tapering or temporarily discontinuing agents is often necessary, particularly corticosteroids, which are linked to poorer outcomes. Biologics are usually withheld until infection control is achieved (Navaneethan et al., 2010). Gut-selective therapies like vedolizumab may be considered in high-risk patients, as they offer effective IBD control with less systemic immunosuppression (Feagan et al., 2013).

### 6.3 Increased risk of complications and need for multidisciplinary management

IBD patients with severe disease are highly vulnerable to CDI complications. Which can progress rapidly and require close monitoring (Debast et al., 2014). Optimal management often involves a team of gastroenterologists, infectious disease specialists and surgeons to coordinate therapy, track complications and determine the need for surgery (Torres et al., 2020). Early surgical consultation is considered in severe and complicated CDI cases to allow timely, life-saving intervention (Siddiqui and Young, 2020).

### 6.4 Potential drug–drug interactions

Managing CDI in IBD patients is complicated by potential drug–drug interactions (DDIs) between the different classes of medications that can affect both efficacy and safety (Khanna and Pardi, 2012). Combining antibiotics, immunosuppressants, biologics and supportive therapies may alter drug absorption and metabolism, increasing the risk of adverse events and complicating treatment. Recognizing and managing these interactions is essential for optimizing outcomes, preventing complications and ensuring patient safety during complex therapy regimens (Debast et al., 2014; Torres et al., 2020; Khanna and Pardi, 2012). Some common drug–drug interactions in CDI and IBD management are listed in Table 1, along with their mechanisms and clinical relevance.

**Table 1:** Common drug–drug interactions in CDI and IBD management require monitoring to prevent adverse effects and optimize treatment outcomes.

Interaction duo	Description	Clinical considerations	References
Vancomycin and immunosuppressants	Minimal systemic absorption generally limits interaction; a compromised gut barrier may increase systemic exposure, causing additive toxicity	Monitor for toxicity in renal impairment and bone marrow suppression risk	Rakočević et al. (2024)
Corticosteroids and metronidazole	CYP450 Enzyme interaction may alter metronidazole levels; Risk of neurotoxicity increased, especially with prolonged corticosteroid use	Monitor neurological symptoms	Hutson et al. (2013) and Debast et al. (2014)
Thiopurines and antibiotics	Some antibiotics can increase thiopurine toxicity, raising bone marrow suppression risk	CBC monitoring; cautious polypharmacy	Singh et al. (2021)
PPIs and CDI antibiotics	PPIs reduce gastric acid, promoting *C. difficile* survival; may lower efficacy of CDI antibiotics	Regularly reassess need of PPI therapy	Deshpande et al. (2012) and Janarthanan et al. (2012)

### 6.5 Interactions between antibiotics and IBD therapies

**Figure 2:**
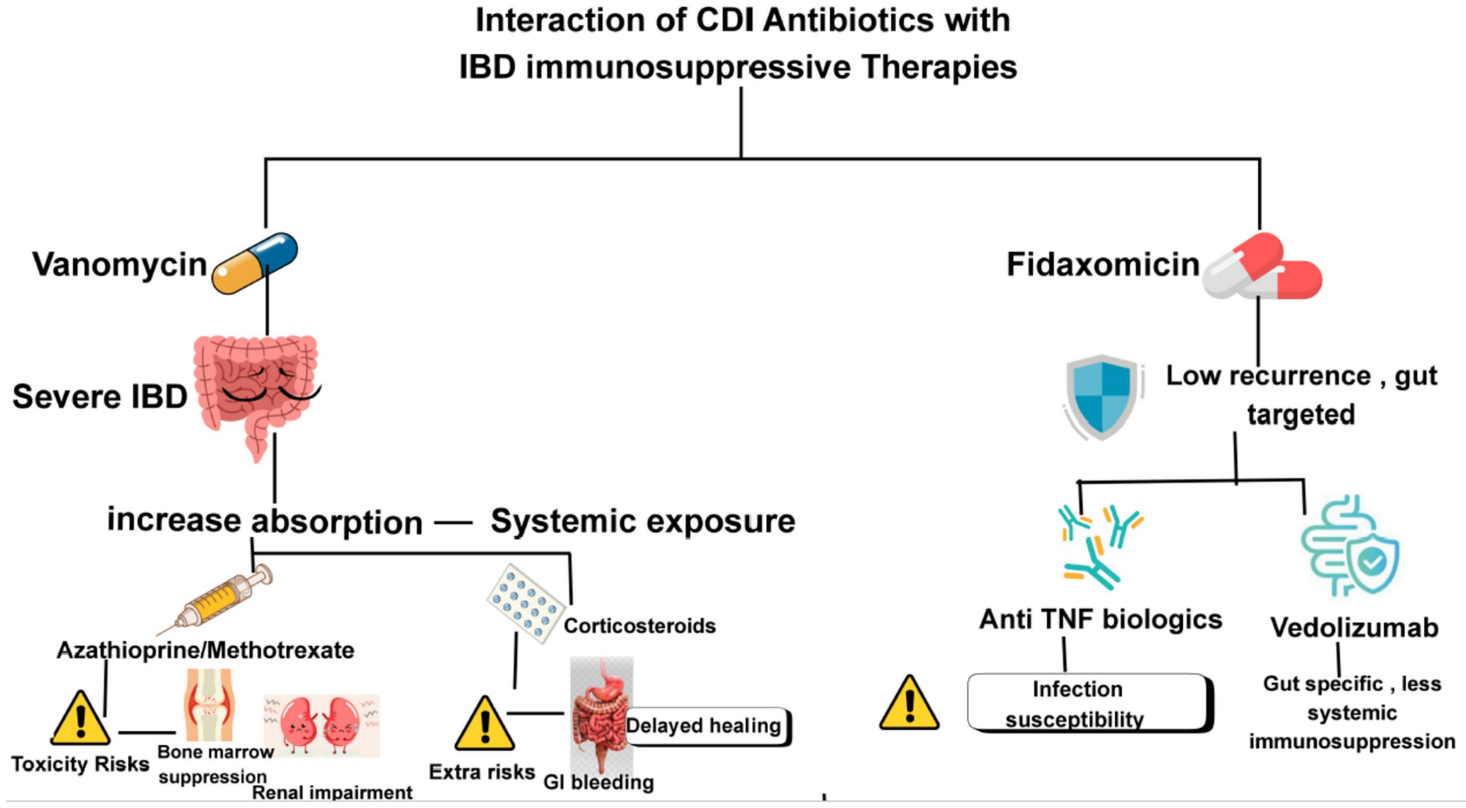
The role of vancomycin and fidaxomicin given with therapies in inflammatory bowel disease.

Oral vancomycin, a first-line CDI antibiotic, is minimally absorbed systemically, limiting most drug interactions. However, in severe IBD with a compromised gut barrier, absorption can increase, rarely leading to systemic exposure and potential additive toxicity when combined with immunosuppressants like azathioprine or methotrexate, especially in patients with renal impairment or bone marrow suppression risk. While direct interactions with corticosteroids are minimal, high-dose steroids with vancomycin may heighten risks of GI bleeding, delayed healing, or reduced CDI treatment response due to immunosuppression (Lei et al., 2019). Fidaxomicin, also minimally absorbed, offers a lower recurrence rate for CDI and less disruption of gut flora. No direct pharmacokinetic interactions exist between biologics (e.g., anti-TNF agents), but combined immune modulation can influence the risk of infection and the course of IBD. Anti-TNF drugs may increase susceptibility to infections, while fidaxomicin could indirectly affect IBD activity by altering the microbiota (Figure 2).

Vedolizumab, with its gut-specific action, offers less systemic immunosuppression and is considered safer in IBD patients at high CDI risk. Clinicians should closely monitor for infection or IBD flares and adjust immunosuppressive regimens as needed during CDI therapy (Cornely et al., 2012).

### 6.6 Interactions between immunosuppressants and CDI antibiotics

Corticosteroids, frequently used in IBD flares, can interact with metronidazole, a treatment for CDI, by affecting cytochrome P450 enzymes that metabolize metronidazole. This interaction may alter drug levels, leading to reduced efficacy or increased risk of toxicity, including neurotoxicity (peripheral neuropathy), especially with long-term corticosteroid use. Patients receiving both should be monitored for neurological symptoms (Hutson et al., 2013; Debast et al., 2014). Another immunosuppressant, Thiopurines (azathioprine, 6-mercaptopurine) are common immunosuppressants for IBD, metabolized via the thiopurine methyltransferase (TPMT) pathway. Some antibiotics, notably allopurinol, although not used for CDI treatment, can interact with thiopurines, heightening bone marrow suppression risk. While metronidazole does not directly interact, combining it with other drugs like allopurinol increases the chance of myelosuppression from toxic metabolic accumulation. Co-administration in polypharmacy situations requires caution (Lim and Chua, 2018). Also both thiopurines and metronidazole can contribute to bone marrow suppression, especially in patients with liver dysfunction or multiple immunosuppressants. Regular complete blood count (CBC) monitoring is essential, and any sign of cytopenia should prompt a review and possible adjustment of concurrent therapies to minimize hematologic risk (Debast et al., 2014).

### 6.7 Interactions involving supportive medications

Proton pump inhibitors (PPIs) used for gastroesophageal reflux disease (GERD) or gastritis in IBD, can raise CDI risk by suppressing gastric acid, weakening the natural defense against pathogens. When combined with CDI antibiotics, such as vancomycin or fidaxomicin, PPIs may further promote *C. difficile* persistence, potentially reducing treatment efficacy. Clinicians should routinely reassess the need for PPIs and consider deprescribing or using the lowest effective dose to minimize the risk (Deshpande et al., 2012; Janarthanan et al., 2012). Probiotics are sometimes added to lessen antibiotic-associated diarrhea or CDI, but their role in IBD is controversial. There are apprehensions that some strains may unpredictably alter the gut microbiome, with possible effects on IBD activity and antibiotic effectiveness. For IBD patients with mild disease and no immunosuppression, certain probiotics, such as *Saccharomyces boulardii*, may be beneficial. However, in those with severe disease or on immunosuppressants, probiotics carry a risk of serious infections (e.g., fungemia) and should be used cautiously or avoided (Klarin et al., 2008).

### 6.8 Interactions affecting absorption and bioavailability

Inflammatory changes, strictures, or past surgeries commonly alter gut absorption in IBD patients. Antibiotics like vancomycin and fidaxomicin, which work locally in the colon, maybe less effective during severe flares due to rapid transit and reduced mucosal area (Khanna and Pardi, 2012). Ongoing inflammation and damage in severe IBD may cause malabsorption, requiring higher or more frequent doses of CDI antibiotics to maintain effective colonic concentrations (Debast et al., 2014). Dosing should be adjusted based on IBD severity and clinical response. Extended regimens, higher doses, or alternative delivery methods such as rectal vancomycin enemas may be needed for optimal treatment (Cornely et al., 2012).

### 6.9 Resistance patterns and treatment failures

CDI in IBD patients poses challenges due to antibiotic resistance and treatment failures. Frequent antibiotic use and immunosuppressive therapies increase the complexity of CDI management. Resistance to standard treatments, recurrent infections, and failures significantly impact outcomes (Markantonis et al., 2024).

#### 6.9.1 Mechanisms of antibiotic resistance in *Clostridioides difficile*

**Figure 3:**
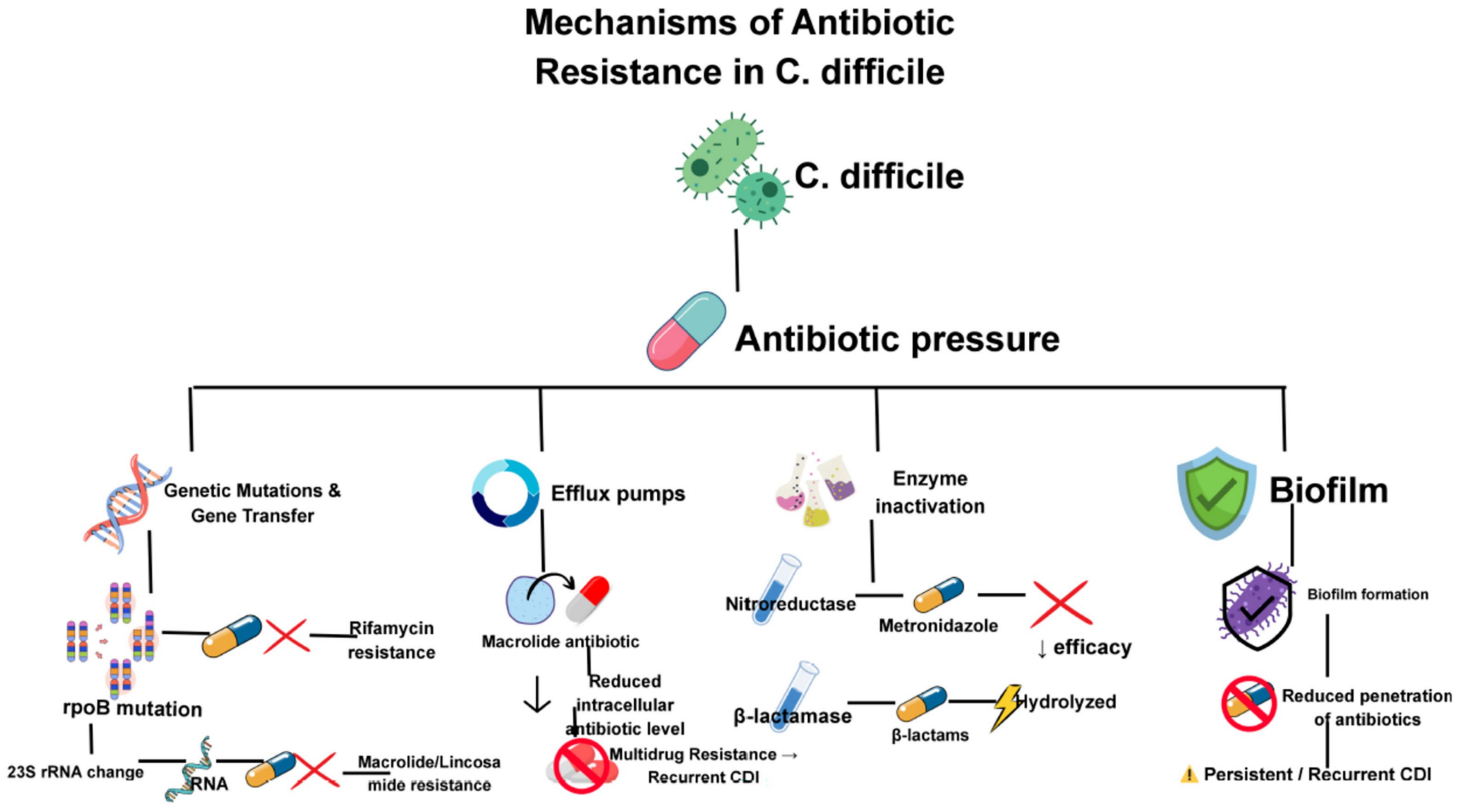
Various mechanisms by which *C. difficile* confers resistance toward antibiotics.

Antibiotic resistance in *C. difficile* complicates CDI treatment, especially in IBD patients frequently exposed to multiple antibiotic courses. Key resistance mechanisms are shown in Figure 3.

Resistance can arise from genetic mutations or horizontal acquisition of resistance genes, i.e., gene transfer on chromosomal DNA or mobile elements like plasmids. For example, rpoB mutations confer rifamycin resistance; 23S rRNA changes cause macrolide and lincosamide resistance (He et al., 2014). Efflux Pumps can actively expel antibiotics such as macrolides, fluoroquinolones, and tetracyclines, lowering intracellular drug levels and contributing to multidrug resistance, complicating recurrent CDI management (Spigaglia, 2016). Some strains produce enzymes that deactivate antibiotics, for example, nitroreductases affecting metronidazole efficacy and beta-lactamases hydrolyzing beta-lactams (Czepiel et al., 2019). *C. difficile* can also form protective biofilms on inflamed mucosa, impeding antibiotic penetration and host immunity, fostering persistent or recurrent infection. This is especially pproblematic in IBD patients due to mucosal damage, promoting biofilm development and resistance (Brandenburg et al., 2015).

#### 6.9.2 Impact of antibiotic resistance patterns on treatment outcomes

Antibiotic-resistant *C. difficile* strains adversely affect treatment outcomes, especially in IBD patients prone to severe or recurrent CDI. It is increasingly being reported that metronidazole resistance leads to higher treatment failure and recurrence rates. IBD patients exposed to repeated metronidazole courses are particularly vulnerable, often requiring alternative therapies (Czepiel et al., 2019). Vancomycin-resistant *C. difficile* (VRCD) is rare, but reduced susceptibility occurs. These strains may need higher doses or longer treatments, especially in IBD patients with extensive colonic disease or altered drug absorption due to rapid transit. Failure often correlates with severe disease and inadequate colonic drug levels (He et al., 2014). Fidaxomicin Resistance is rare due to its unique RNA polymerase target, but emerging cases raise concerns, as it is critical in managing recurrent CDI in IBD patients (Cornely et al., 2012). Fluoroquinolone-resistant strains, such as the BI/NAP1/027 epidemic hypervirulent strain produce more toxins, show high fluoroquinolone resistance, and cause more severe disease and recurrences. IBD patients infected with such strains face worsened outcomes and need aggressive management (Loo et al., 2005; Kuijper et al., 2006).

Treatment failures in IBD patients with CDI result from resistant strains, altered antibiotic pharmacokinetics, and host factors. Repeated antibiotic courses for infections or surgeries in IBD patients promote resistant *C. difficile* strains and disrupt gut microbiota, increasing recurrence risk. Frequent hospitalizations and immunosuppression further drive resistance (Slimings and Riley, 2014). Immunosuppressive medications impair the immune clearance of *C. difficile*, thereby heightening the risk of severe and recurrent infections. They may also alter antibiotic metabolism, reducing effectiveness (Ananthakrishnan et al., 2013). Chronic inflammation, mucosal ulceration, and dysbiosis in IBD lead to rapid intestinal transit and mucosal damage, limiting antibiotic concentration and penetration at infection sites, contributing to treatment failure (Seekatz et al., 2014). Biofilm-associated *C. difficile* in severe IBD protects bacteria from antibiotics and immune attack, fostering persistent infection and resistance. Recurrent CDI increases morbidity and complicates management in this population (Dapa and Unnikrishnan, 2013).

### 6.10 Strategies for managing treatment failures

Effective management of treatment failures in IBD patients with CDI requires addressing both infection and underlying IBD. Optimization of antibiotic therapy is crucial. Using combination therapy (e.g., oral vancomycin plus IV metronidazole) in severe cases may be necessary. Higher or extended dosing can help achieve therapeutic levels in patients with severe colonic inflammation or altered absorption (Cornely et al., 2012). Fidaxomicin use can be beneficial in recurrent CDI due to its narrow spectrum and minimal microbiota disruption (Louie et al., 2011). FMT may restore gut flora and may be effective for recurrent CDI after multiple antibiotic failures. In IBD, FMT carries flare risk and requires careful monitoring (Paramsothy et al., 2017; Quraishi et al., 2017). Monoclonal antibodies like bezlotoxumab targeting *C. difficile* TcdB can reduce recurrence, especially in high-risk IBD patients (Wilcox et al., 2017). Optimizing IBD treatment is crucial to decrease CDI recurrence. Modifying immunosuppressants or biologics and balancing inflammation control without excessive immunosuppression is essential (Ananthakrishnan et al., 2013). Minimizing unnecessary antibiotics through stewardship and alternative IBD management helps prevent resistance and treatment failures (Slimings and Riley, 2014). The various therapeutic strategies for CDI treatment and their stage of study have been discussed in Table 2.

**Table 2:** Therapeutic strategies for CDI in IBD patients.

Strategy	Type of study	Description	References
Fidaxomicin vs. vancomycin	Clinical	Comparative trials showing fidaxomicin’s comparable cure rates and significantly lower recurrence vs. vancomycin	Louie et al. (2011) and Cornely et al. (2012)
FMT	Clinical	Studies demonstrating high efficacy of FMT for recurrent CDI in IBD patients, with variable IBD flare outcomes	Fischer et al. (2016), Kelly et al. (2014), Cammarota et al. (2015), and Allegretti et al. (2020)
Vaccine	Clinical	Advanced clinical trials of toxoid-based vaccines targeting *C. difficile* toxins; evaluation of efficacy and safety in immunocompromised (IBD) patients	Wilcox et al. (2017)
Probiotics	Clinical	Meta-analyses and randomized controlled trials assessing probiotics’ effect on antibiotic-associated diarrhea and CDI in general	Goldenberg et al. (2017)
Antibiotic resistance mechanisms in *C. difficile*	Pre-clinical and clinical	Studies investigating genetic mutations, efflux pumps, enzyme inactivation, and biofilm formation related to antibiotic resistance	He et al. (2014), Brandenburg et al. (2015), and Spigaglia (2016)
Bezlotoxumab	Clinical	Reduces CDI recurrences, including in high-risk IBD patients	Wilcox et al. (2017)
Microbiome-based and Bacteriophage therapies	Pre-clinical and early clinical	Evaluations of live biotherapeutics, bacteriophage therapy and microbiota-based drugs for microbiome modulation and CDI prevention	Buffie et al. (2014) and Khanna et al. (2016)
Predictive models and personalized medicine	Clinical/pre-clinical	Use of clinical data, genetics and machine learning for risk stratification and targeted preventive strategies in IBD patients at risk for CDI	Mahnic et al. (2022) and Bai et al. (2023)

## 7 Preventive strategies for CDI in IBD patients

### 7.1 Antimicrobial stewardship

Antimicrobial stewardship involves coordinated strategies to optimize antibiotic use, aiming to treat infections effectively while minimizing harms like resistance, toxicity and gut microbiota disruption. In IBD patients, stewardship is especially important because frequent antibiotic use for infections or complications increases their risk of CDI and recurrence (Slimings and Riley, 2014). Applying stewardship principles helps reduce CDI incidence, improve outcomes and preserve antibiotic effectiveness. This section outlines the key goals and strategies of antimicrobial stewardship tailored to IBD patients at risk for CDI. The primary goal of antimicrobial stewardship in IBD patients is to optimize antibiotic use for the best outcomes while minimizing the risks from inappropriate or excessive exposure. Key principles include appropriately choosing antibiotics. Choosing targeted agents based on infection type, patient factors, local resistance, and microbiota impact, favoring narrow-spectrum antibiotics to preserve gut flora and reduce CDI risk (Crobach et al., 2016). Tailoring dose and length to maximize efficacy and limit adverse effects or resistance, considering altered pharmacokinetics, prior antibiotic use, and CDI risk in IBD. It is necessary to restrict antibiotics to clear bacterial infections, avoiding them for viral illnesses, mild IBD flares or uncomplicated conditions where benefits are unclear (Davey et al., 2017). Continuous monitoring of reassessing therapy, using culture data and patient response to guide narrowing or stopping antibiotics to reduce unnecessary exposure (Baur et al., 2017).

Effective antimicrobial stewardship in IBD patients at risk for CDI involves multiple strategies, including the development of evidence-based, locally tailored protocols for antibiotic use in IBD, including standardized approaches to diagnosis, treatment choice, and duration (Slimings and Riley, 2014). Providing targeted recommendations for common IBD infections (e.g., perianal abscess, pouchitis), favoring appropriate agents like short-course metronidazole and ciprofloxacin, and avoiding high-risk antibiotics for non-specific symptoms (Slimings and Riley, 2014). Regularly auditing antibiotic prescribing patterns to detect inappropriate use and provide prescribers with feedback and education on stewardship principles and CDI risk reduction (Davey et al., 2017). Promoting the use of narrow-spectrum antibiotics when possible to preserve gut microbiota and reduce CDI risk. Encourage alternatives to high-risk drugs like clindamycin and fluoroquinolones, substituting safer options such as doxycycline when appropriate (Czepiel et al., 2019). Preferring shorter antibiotic courses (e.g., 5–7 days) has been shown to be equally effective and less likely to cause CDI; individualize duration based on clinical response and reassess need frequently (Spellberg and Rice, 2019). Limiting *C. difficile* testing to clinically warranted cases, avoiding routine screening that may detect asymptomatic colonization. Employ rapid diagnostics (e.g., PCR) alongside clinical assessment to guide appropriate therapy, preventing overtreatment (Crobach et al., 2016). Implementing antimicrobial stewardship in IBD patients offers several benefits including reduced incidence of CDI. Optimizing antibiotic use reduces exposure to high-risk agents, such as fluoroquinolones and clindamycin, thereby preserving gut microbiota and decreasing the risk of CDI (Baur et al., 2017). The appropriate antibiotic use reduces CDI complications, such as toxic megacolon, sepsis, and colectomy, while minimizing adverse drug reactions and enhancing the quality of life (Reznik et al., 2025). Stewardship slows resistance development by promoting judicious use of antibiotics, critical for IBD patients frequently receiving multiple antibiotic courses (Davey et al., 2017). By preventing unnecessary antibiotic use and CDI-related complications, stewardship programs reduce hospitalizations, extended treatments and surgical costs, easing healthcare financial burdens (Baur et al., 2017). While antimicrobial stewardship is beneficial, its use in IBD patients faces challenges such as frequent hospitalizations and infections require careful balancing of necessary antibiotic use with stewardship principles, which is especially tough in severe cases (Slimings and Riley, 2014). IBD flares and infections share symptoms, making it hard to know when antibiotics are needed and increasing the risk of overuse. Patients and providers may resist limiting antibiotics due to safety concerns, so education is crucial. Effective programs demand staff, diagnostics, and time, which may be lacking in smaller or outpatient settings.

### 7.2 Probiotics and prebiotics

Probiotics and prebiotics are being explored as adjuncts to prevent and manage CDI in IBD patients, who are at increased risk due to disrupted gut microbiota, frequent antibiotic use and immunosuppression. They aim to restore healthy microbiota and improve resistance to *C. difficile*, but their effectiveness and safety, especially in IBD, are still debated and complex (Singh et al., 2025).

#### 7.2.1 Mechanisms of action of probiotics in the treatment of CDI in IBD patients

Probiotics are live microorganisms that, when taken in sufficient amounts, provide health benefits by restoring gut microbial balance and helping the body resist pathogens. Common strains include Lactobacillus, Bifidobacterium, *Saccharomyces boulardii*, and *Streptococcus*. Mechanistically, probiotics replenish beneficial bacteria lost due to antibiotics or disease, compete with harmful microbes for nutrients and space, and produce antimicrobial compounds, such as organic acids and bacteriocins, that inhibit pathogens, including *C. difficile*. They also enhance the intestinal barrier by increasing mucus production and tight junction proteins, thereby reducing the translocation of harmful bacteria. Additionally, probiotics modulate immunity by promoting anti-inflammatory cytokines and regulatory T cells, helping reduce inflammation and aid mucosal healing, especially important for IBD and CDI patients (Latif et al., 2023).

Probiotics are widely explored for CDI prevention in high-risk groups, including those on antibiotics, yet evidence for their benefit in IBD patients is inconsistent. Some clinical studies and meta-analyses suggest strains like *Saccharomyces boulardii* and *Lactobacillus rhamnosus* GG can reduce rates of antibiotic-associated diarrhea and CDI in general, but these findings may not extend reliably to IBD patients due to varied study designs and populations. In IBD, some research reports lower CDI rates with probiotics, while others show no significant effect. Heterogeneity in probiotic strain, dose and patient selection fuels uncertainty. Probiotics are also considered as adjuncts in treating active and recurrent CDI, yet evidence for their effectiveness in IBD is limited and conflicting. Some trials report fewer CDI recurrences with added probiotics, while others show no benefit or highlight risk, such as fungemia with *S. boulardii*, especially in immunocompromised individuals. Thus, guidelines generally do not recommend routine probiotic use for CDI in IBD until more robust data are available (Goldenberg et al., 2017).

Probiotics use in IBD carries risks that require careful consideration. Immunocompromised IBD patients, especially those on immunosuppressive drugs, face increased risks of infections such as bacteremia, sepsis, and fungemia, particularly with strains like *Saccharomyces boulardii* and some Lactobacillus species. Probiotics vary widely in strain, doses and formulation, making their efficacy unpredictable. Certain probiotics could potentially worsen IBD symptoms, with some reports linking *Lactobacillus rhamnosus* to flare-ups, underscoring the need for cautious use and monitoring. Additionally, probiotics are often sold as dietary supplements without stringent regulation, leading to variable quality, potency, and purity. This lack of standardization complicates ensuring consistent safety and effectiveness. Overall, while probiotics may offer benefits, their use in IBD should be personalized and supervised by healthcare professionals to minimize risks and optimize outcomes (Sanders et al., 2013).

#### 7.2.2 Mechanism of action of prebiotics in the treatment of CDI in IBD patients

Prebiotics are non-digestible food components, mainly dietary fibers or complex carbohydrates like inulin, fructooligosaccharides (FOS), and galactooligosaccharides (GOS), that selectively stimulate the growth or activity of beneficial gut bacteria. They support gut health by promoting beneficial bacterial growth, enhancing colonization resistance against harmful pathogens, and producing SCFAs such as butyrate, which have anti-inflammatory and immune-modulating effects. In patients with IBD, prebiotics may help restore balanced gut microbiota and reduce inflammation, potentially lowering the risk of CDI. The fermentation of prebiotics by gut bacteria is key to these benefits, as it fuels the production of SCFAs and supports a healthier intestinal environment. Overall, prebiotics contribute to gut microbial balance and immune health, playing a supportive role in managing intestinal disorders (Koh et al., 2016).

There is limited direct evidence on prebiotics for preventing CDI in IBD patients. Some studies indicate prebiotics may promote beneficial bacteria like Bifidobacterium and Lactobacillus, which inhibit *C. difficile* growth. However, their role in treating active CDI is less clear. Prebiotics might support gut health but could worsen symptoms like gas and distension in active colitis. Overall, prebiotics’ effectiveness and safety for CDI prevention and treatment in IBD remain uncertain and require further research (Ríos-Covián et al., 2016). Careful patient selection is key when using probiotics or prebiotics in IBD. Those with mild to moderate disease and no severe immunosuppression are better candidates. Patients with severe disease, active players, or significant immunosuppression face higher risks and need close monitoring. Choosing specific probiotic strains with evidence for CDI prevention or treatment, like *Saccharomyces boulardii* or *Lactobacillus rhamnosus GG*, is advisable. Using products from reputable sources ensures quality and safety. Monitoring for adverse effects like GI symptoms, infections, or IBD exacerbations is essential for early detection and management. Regular follow-up supports patient safety and treatment success (Akutko and Stawarski, 2021).

As CDI cases rise in IBD patients, tailored prevention is crucial. Current strategies like antimicrobial stewardship, probiotics and prebiotics provide some benefits but have limitations. Future approaches may combine novel therapies, better diagnostics, personalized risk assessments, and advanced treatments to improve CDI prevention in this high-risk group (Smits et al., 2016).

### 7.3 Vaccination against *Clostridioides difficile*

Vaccination presents a promising approach for CDI prevention, especially in high-risk groups like IBD patients. Current vaccines target *C. difficile* toxins, TcdA and TcdB to generate neutralizing antibodies, aiming to prevent or lessen disease severity. Toxoid-based vaccines utilize inactivated toxins, demonstrating a strong immune response in early trials; however, their effectiveness in IBD patients, who often have altered immunity, remains unclear. Several candidates, including Pfizer’s PF-06425090, are in advanced clinical trials assessing prevention of primary and recurrent CDI. Challenges specific to IBD patients include immunosuppressive therapy potentially reducing vaccine efficacy, safety concerns regarding IBD symptom exacerbation, and unknown long-term immunity duration. Further research is needed to determine the optimal vaccination timing, safety, and whether booster doses are necessary to maintain protection in this population. Despite setbacks in meeting primary endpoints, ongoing vaccine development continues to offer hope for the prevention of CDI in vulnerable groups (Wilcox, 2012).

### 7.4 Targeted microbiome modulation

The gut microbiome is vital for intestinal health and protection against CDI. In IBD patients, dysbiosis, an imbalance in gut microbiota, raises CDI risk. Microbiome modulation aims to restore a healthy microbial community that resists colonization *by C. difficile*. Emerging therapies include live biotherapeutics using beneficial bacterial strains like *Faecalibacterium prausnitzii* and *Akkermansia muciniphilia*, which show anti-inflammatory effects and support the gut barrier. Clinical trials are evaluating their safety and efficacy in IBD patients (Buffie et al., 2014). Bacteriophage therapy employ viruses that specifically target and kill *C. difficile* without harming beneficial microbes. This approach, alone or with antibiotics, has shown promise in animal studies, with human trials planned (Khanna et al., 2016). Additionally, microbiota-based drugs such as defined microbial consortia offer a controlled alternative to FMT. These combinations of live microbes aim to restore gut balance and prevent CDI more predictably than FMT, which can have variable effects. Together, these innovative strategies hold potential to improve CDI prevention and gut health in IBD patients by targeting microbial imbalances and enhancing colonization resistance.

### 7.5 Improved diagnostic tools for early detection

Early, accurate diagnosis of CDI in IBD patients is essential to prevent severe disease and transmission. Current tests like stool toxin assays and molecular methods face challenges due to IBD symptoms overlap. Advances include NGS, which profiles gut microbiome changes preceding infection, enabling earlier intervention. Rapid POC tests detecting *C. difficile* toxins provide quick and sensitive bedside diagnosis, crucial for timely management of IBD patients. Biomarker-based assays aimed at differentiating CDI from IBD flares utilize host immune markers, metabolites, or microbial components. Combining these biomarkers could enhance diagnostic accuracy and inform treatment decisions. Together, these emerging technologies promise faster, more precise CDI detection in IBD, improving patient outcomes by enabling tailored therapies and reducing misdiagnosis (Polage et al., 2015).

### 7.6 Personalized risk assessment and predictive models

Personalized medicine in CDI prevention tailor strategies to individual IBD patients by assessing genetic susceptibility, comorbidities, medications and microbiome composition. Predictive models that incorporate clinical data, laboratory markers, and advanced analytics, such as machine learning, help identify high-risk patients. These models, integrated into clinical decision systems, guide providers in selecting targeted preventive measures such as prophylactic antibiotics, adjusting immunomodulators or early use of probiotics or vaccines. This approach enhances the effectiveness of prevention by focusing on patient-specific risks and optimizing therapy decisions. As a result, personalized medicine holds promise for improving CDI outcomes and management in the heterogeneous IBD population (Ashton et al., 2019). Table 3 summarizes future and emerging CDI preventive strategies tailored for IBD patients, emphasizing the promise and challenges of innovative approaches beyond traditional measures.

**Table 3:** Emerging preventive strategies for CDI in IBD patients.

Strategy	Explanation	Challenges	References
Antimicrobial stewardship	Optimize antibiotic use to preserve gut microbiota and reduce CDI risk	Requires institutional support, education, diagnostic clarity and resource allocation	Baur et al. (2017)
Probiotics and prebiotics	Restore gut microbial balance and enhance colonization resistance; potential to reduce CDI incidence	Efficacy in IBD uncertain; risk of infections in immunocompromised; strain and dose variability	Goldenberg et al. (2017)
Vaccination against *C. difficile*	Vaccines targeting toxins to generate protective immunity; candidates under clinical trials	Immunocompromised patient response, safety concerns, long-term efficacy and timing of vaccination	Wilcox (2012)
Microbiome modulation	Use of targeted live biotherapeutics, bacteriophages, and microbiota-based drugs to restore healthy gut flora	Experimental; safety and efficacy in IBD populations need validation	Buffie et al. (2014) and Khanna et al. (2016)
Personalized risk assessment and predictive modeling	Use of genetics, clinical data, microbiome profiles and machine learning to identify high-risk patients and tailor prevention	Requires large data sets, integration into clinical workflows and ongoing validation	Singh (2019)

## 8 Novel therapeutic approaches

Monoclonal antibodies like bezlotoxumab target *C. difficile* TcdB and reduce CDI recurrence in high-risk patients, including those with IBD. While bezlotoxumab is effective alone, combining it with FMT has shown no clear added benefit. Future research may optimize the use of antibodies by combining them with vaccines or microbiome therapies. Immune modulation therapies, such as checkpoint inhibitors and cytokine blockers, would enhance host defenses but require caution to avoid worsening IBD inflammation. Emerging CRISPR-CAS9 gene-editing therapies offer potential to disable antibiotic resistance and virulence genes in *C. difficile*, potentially reducing resistant infections and improving antibiotic efficacy. Although promising, these approaches are in early stages and need more research for safety and effectiveness in IBD populations (Bikard et al., 2014).

## 9 Implementation of integrated preventive programs

Multidisciplinary and collaborative approaches: Effective CDI prevention in IBD patients necessitates a coordinated, multidisciplinary approach. Success relies on collaboration among gastroenterologists, infectious disease experts, microbiologists, pharmacists, nurses and often dietitians and psychologists to address both infection control and chronic disease management. This approach improves identification of high-risk individuals, optimizes preventive interventions, and ensures continuous reassessment and rapid adaptation of strategies during patient care (Doll et al., 2021; Tang et al., 2025). Patient education and empowerment: Patient education is a cornerstone of CDI prevention in IBD care. Well-informed patients can take a proactive role in recognizing early symptoms of CDI and seeking prompt care; understanding the risks of indiscriminate antibiotic use and adhering to stewardship advice; following recommended preventive measures; actively participating in schedule checkups, reporting changes in symptoms and compliance with infection control protocols; utilizing available education materials, support groups provided by multidisciplinary team to stay informed and engaged with car. Areas in need of exploration include improved bikerse plan (Khanna, 2020a; Doll et al., 2021; Raffaele et al., 2024; Singh et al., 2024). Empowering patients in this way leads to better health outcomes, reduced recurrence rates and improved adherence to both preventive and therapeutic regimens (Doll et al., 2021; Raffaele et al., 2024).

## 10 Future directions and research gaps

Despite advances, management of CDI in IBD presents key research gaps, including ongoing diagnostic ambiguities, variable treatment responses and high recurrence rates. The overlapping clinical features of CDI and IBD flares, along with the impact of frequent antibiotics and biologics, highlight the urgent need for strategies that go beyond standard algorithms. Areas in need of exploration include improved biomarkers and rapid diagnostics, refinement of integrated prevention programs, and a deeper understanding of the long-term safety of novel therapies, such as live microbiota products and CDI vaccines (Alhobayb and Ciorba, 2023; Bai et al., 2023).

### 10.1 Need for personalized medicine approaches

Given the heterogeneity of both IBD and CDI, personalized medicine, taking into account a patient’s clinical phenotype, genetics, microbiome, and immune status, is critical for optimizing care. Individualized approaches can help stratify risk, guide antibiotic and immunosuppressive stewardship, and support tailored patient monitoring, thus potentially improving outcomes. Advanced predictive models and risk calculators, powered by patient-specific data, can help clinicians pre-emptively identify patients at the highest risk for CDI and recurrence, as well as those who are most likely to benefit from emerging vaccine or microbiota therapies (Mahnic et al., 2022; Alhobayb and Ciorba, 2023; Bai et al., 2023).

### 10.2 Development of novel diagnostic and therapeutic tools

Emerging diagnostic technologies, such as NGS, multi-pathogen PCR panels, cytokine profiles, and metabolite-based biomarker assays, are being developed to improve the rapid and accurate discrimination of CDI from IBD flares. Meanwhile, innovative therapies under investigation include defined microbial consortia, targeted live biotherapeutics, phage therapy, monoclonal antibodies, and CDI vaccines. Despite early promise, these tools required rigorous validation specifically in IBD populations to assess their true clinical value, safety, and long-term effects (Bai et al., 2023; Sangurima et al., 2023).

### 10.3 Understanding host-microbiome interactions in IBD patients with CDI

The host-microbiome interaction is fundamental in CDI pathogenesis and recurrence, particularly in the context of IBD, where dysbiosis and immune dysfunction often coincide. Research shows that both IBD and CDI are characterized by reduced microbial diversity, expansion of pathogenic bacteria and impaired colonization resistance, which together exacerbate inflammation and infection risk. Further studies must focus on mechanistic, longitudinal analyses of microbiota composition, host immune responses and the effects of interventions like immunosuppressants, FMT or emerging microbiome-directed therapies in various IBD phenotypes (Mahnic et al., 2022; Alhobayb and Ciorba, 2023).

## 11 Conclusion

CDI in IBD is a growing problem, marked by frequent diagnostic challenges, recurrences, and adverse outcomes due to overlapping symptoms, immunosuppressive therapy, and the prevalence of gut dysbiosis. Standard antibiotics remain the cornerstone of CDI management, with fecal microbiota transplantation (FMT) and enhanced preventive strategies becoming increasingly important in patients with IBD. However, further refinement of management protocols is needed, particularly for recurrent CDI and in the context of complex immunosuppressive regimens (Bai et al., 2023). The key clinical take-home messages are: (1) clinicians must maintain a high index of suspicion for CDI in IBD patients presenting with recurrent or worsening diarrhea, regardless of recent antibiotic exposure; and (2) optimal outcomes require a multidisciplinary, personalized approach that integrates stepwise diagnostics, microbiota assessment, and tailored therapeutic strategies. Close collaboration among gastroenterology, infectious diseases, pharmacy, and microbiology teams, alongside proactive patient education, is essential to reduce morbidity and improve quality of life in this high-risk population (Khanna, 2020b; Alhobayb and Ciorba, 2023).
